# Supplementary material for: Impact of etonogestrel implant use on T-cell and cytokine profiles in the female genital tract and blood
Source: PLoS One. 2020 Mar 26;15(3):e0230473. doi: 10.1371/journal.pone.0230473 (PMC7098611; doi:10.1371/journal.pone.0230473)
Supplement: S1 Appendix — (DOCX) [file pone.0230473.s001.docx]

| **Appendix Table 1. Estimated cytokine levels in the CVL for Implant users, adjusting for repeated measures and covariates** | | | | | | | | | |
| --- | --- | --- | --- | --- | --- | --- | --- | --- | --- |
|  | **Pre-Contraceptive** | | | **Post-Contraceptive** | | | **Arithmetic Mean Ratio Post:Pre** | | |
| **Cytokine** | **Estimate*** | **95% CI LB*** | **95% CI UB*** | **Estimate*** | **95% CI LB*** | **95% CI UB*** | **AMR** | **95% CI LB** | **95% CI UB** |
| *INFa2* | 27.55 | 17.19 | 44.14 | 22.72 | 14.01 | 36.83 | 0.82 | 0.61 | 1.11 |
| *INFg* | 9.30 | 4.67 | 18.52 | 6.54 | 3.16 | 13.57 | 0.70 | 0.41 | 1.20 |
| *IL2* | 10.10 | 5.42 | 18.79 | 7.23 | 3.85 | 13.60 | 0.72 | 0.52 | 0.99 |
| *IL4* | 36.47 | 21.56 | 61.67 | 26.94 | 15.63 | 46.42 | 0.74 | 0.50 | 1.09 |
| *IL6* | 81.25 | 34.84 | 189.48 | 57.02 | 24.05 | 135.21 | 0.70 | 0.43 | 1.14 |
| *IL12p70* | 16.13 | 9.39 | 27.73 | 11.19 | 6.44 | 19.43 | 0.69 | 0.51 | 0.94 |
| *IL17* | 3.84 | 2.12 | 6.98 | 4.61 | 2.49 | 8.52 | 1.20 | 0.80 | 1.80 |
| *IL1a* | 807.71 | 344.66 | 1892.90 | 700.10 | 294.79 | 1662.66 | 0.87 | 0.56 | 1.35 |
| *IL1b* | 191.15 | 86.92 | 420.35 | 121.74 | 54.07 | 274.10 | 0.64 | 0.37 | 1.09 |
| *GCSF* | 1518.70 | 709.47 | 3250.94 | 814.34 | 377.48 | 1756.80 | 0.54 | 0.39 | 0.74 |
| *GMCSF* | 10.56 | 5.73 | 19.46 | 8.16 | 4.38 | 15.21 | 0.77 | 0.55 | 1.08 |
| *TNFa* | 14.98 | 7.63 | 29.41 | 13.10 | 6.59 | 26.04 | 0.87 | 0.60 | 1.27 |
| *sCD40L* | 36.92 | 24.98 | 54.57 | 53.89 | 35.70 | 81.35 | 1.46 | 1.02 | 2.08 |
| *MIP1a* | 36.65 | 16.27 | 82.53 | 33.16 | 14.52 | 75.72 | 0.90 | 0.58 | 1.40 |
| *MIP1b* | 54.81 | 24.67 | 121.73 | 41.60 | 18.58 | 93.13 | 0.76 | 0.54 | 1.06 |
| *IL8* | 3083.99 | 1952.45 | 4871.32 | 2766.11 | 1726.98 | 4430.50 | 0.90 | 0.65 | 1.23 |
| *IP10* | 1576.11 | 807.00 | 3078.22 | 1088.04 | 543.54 | 2177.99 | 0.69 | 0.42 | 1.14 |
| *Fractalkine* | 235.34 | 146.64 | 377.68 | 178.06 | 109.55 | 289.42 | 0.76 | 0.55 | 1.03 |
| Generalized linear mixed model controlling for semen, STI, blood contamination, BV, and a random intercept for participant, variance components covariance structure, gamma distribution, log link | | | | | | | | | |
| * Back-transformed estimate (arithmetic mean) | | | | |  |  |  |  |  |
