# Supplementary material for: Impact of etonogestrel implant use on T-cell and cytokine profiles in the female genital tract and blood
Source: PLoS One. 2020 Mar 26;15(3):e0230473. doi: 10.1371/journal.pone.0230473 (PMC7098611; doi:10.1371/journal.pone.0230473)
Supplement: S2 Appendix — (DOCX) [file pone.0230473.s002.docx]

| **Appendix Table 2: Estimated cytokine levels in the PBMC for Implant users, adjusting for repeated measures** | | | | | | | | |  | |  | | | | |  |  |  |  |
| --- | --- | --- | --- | --- | --- | --- | --- | --- | --- | --- | --- | --- | --- | --- | --- | --- | --- | --- | --- |
|  | **Pre-Contraceptive** | | | **Post-Contraceptive** | | | **Arithmetic Mean Ratio Post:Pre** | | | |  | |  |  |  |  |  |  |  |
| **Cytokine** | | **Estimate*** | **95% CI LB*** | **95% CI UB*** | **Estimate*** | **95% CI LB*** | **95% CI UB*** | **AMR** | **95% CI LB** | **95% CI UB** | | |  | |  |  |  |  |  |
| INFa2 | | 851.58 | 547.78 | 1323.87 | 784.05 | 502.29 | 1223.86 | 0.92 | 0.79 | 1.07 | | |  | |  |  |  |  |  |
| INFg | | 542.65 | 285.22 | 1032.44 | 505.49 | 264.48 | 966.12 | 0.93 | 0.77 | 1.12 | | |  | |  | |  |  |  |
| IL2 | | 22.72 | 10.01 | 51.58 | 28.16 | 12.26 | 64.67 | 1.24 | 0.88 | 1.74 | | |  | |  | |  |  |  |
| IL4 | | 747.65 | 432.64 | 1292.02 | 638.64 | 368.05 | 1108.15 | 0.85 | 0.72 | 1.01 | | |  | |  | |  |  |  |
| IL6 | | 43.76 | 15.40 | 124.32 | 42.20 | 14.79 | 120.42 | 0.96 | 0.76 | 1.22 | | |  | |  | |  |  |  |
| IL12p70 | | 122.98 | 67.41 | 224.35 | 106.84 | 58.20 | 196.13 | 0.87 | 0.70 | 1.08 | | |  | |  | |  |  |  |
| IL17 | | 64.77 | 32.66 | 128.47 | 63.87 | 32.08 | 127.19 | 0.99 | 0.82 | 1.18 | | |  | |  | |  |  |  |
| IL1a | | 16.30 | 4.70 | 56.55 | 16.23 | 4.65 | 56.59 | 1.00 | 0.76 | 1.31 | | |  | |  | |  |  |  |
| IL1b | | 29.46 | 17.48 | 49.63 | 27.14 | 16.01 | 45.99 | 0.92 | 0.76 | 1.12 | | |  | |  | |  |  |  |
| GCSF | | 558.84 | 356.79 | 875.32 | 466.80 | 296.78 | 734.21 | 0.84 | 0.72 | 0.97 | | |  | |  | |  |  |  |
| GMCSF | | 249.73 | 142.66 | 437.16 | 222.29 | 126.43 | 390.85 | 0.89 | 0.75 | 1.06 | | |  | |  | |  |  |  |
| TNFa | | 152.55 | 102.35 | 227.35 | 143.60 | 95.92 | 214.99 | 0.94 | 0.81 | 1.09 | | |  | |  | |  |  |  |
| sCD40L | | 5358.60 | 4080.78 | 7036.55 | 4986.68 | 3779.74 | 6579.02 | 0.93 | 0.82 | 1.06 | | |  | |  | |  |  |  |
| MIP1a | | 6.93 | 2.38 | 20.23 | 5.92 | 2.02 | 17.35 | 0.85 | 0.67 | 1.09 | | |  | |  | |  |  |  |
| MIP1b | | 421.69 | 273.53 | 650.10 | 381.31 | 246.47 | 589.90 | 0.90 | 0.79 | 1.04 | | |  | |  | |  |  |  |
| IL8 | | 2.16 | 0.98 | 4.77 | 2.00 | 0.89 | 4.47 | 0.93 | 0.65 | 1.32 | | |  | |  | |  |  |  |
| IP10 | | 3496.65 | 2278.88 | 5365.16 | 3249.00 | 2113.09 | 4995.54 | 0.93 | 0.84 | 1.03 | | |  | |  | |  |  |  |
| Fractalkine | | 174.59 | 76.89 | 396.43 | 160.19 | 70.42 | 364.36 | 0.92 | 0.80 | 1.05 | | |  | |  | |  |  |  |
| Generalized linear mixed model with random intercept for participant, variance components covariance strutcure, gamma distribution, log link | | | | | | | | | | | | | | | | | | |  |
| * Back-transformed estimate (arithmetic mean) | | | | |  |  |  |  |  | |  | | | | |  | |  | |
