# Supplementary material for: Impact of etonogestrel implant use on T-cell and cytokine profiles in the female genital tract and blood
Source: PLoS One. 2020 Mar 26;15(3):e0230473. doi: 10.1371/journal.pone.0230473 (PMC7098611; doi:10.1371/journal.pone.0230473)
Supplement: Attachment — Submitted filename: Lisa Haddad review for PLOSOne.docx [file pone.0230473.s003.docx]

Lisa Haddad et al. studied the effects of Nexplanon contraceptive implant use on vaginal and systemic immune parameters associated with HIV acquisition. This report is timely due to the current controversy about whether depoprovera (DMPA) and other types of hormonal contraception affect susceptibility to HIV-1 infection. Like DMPA, the active component in Nexplanon, etonogestrel (Eng), is a synthetic progestin that targets the glucocorticoid receptor in addition to the progesterone receptor. This is the first report on the potential effects of Eng on biological factors underlying HIV risk. The investigators used a similar approach to earlier investigations done by others on the effects of DMPA on vaginal and systemic immunity. It should be noted that these studies to date have not reached a consensus on the effects of DMPA on vaginal immunity. *[ Byrne et al (2016) detected increased numbers and frequency of CCR5+ CD4+ T cells in endocervical cytobrush samples from women on DMPA, but detected a similar increase during the luteal phase of the menstrual cycle in women that did not use DMPA. They also reported no significant effect on 14 vaginal cytokine concentrations. Smith-McCune et al (2017) detected no increase in the number or frequency of CCR5+ CD4+ T cells in endocervical cytobrush samples, but found increased concentrations of MCP1 and IFN alpha 2 in endocervical mucus. Mitchell et al (2014) found no effect of DMPA on numbers or frequency of CCR5+ CD4+ cells in ectocervical biopsies. Other large cohort studies have detected higher concentrations of RANTES and other chemokines in vaginal fluid from women on DMPA,]*

This study reports a significant increase in the percent of T cells expressing CCR5 and the central memory phenotype (CCR7) in cells recovered from cervicovaginal lavage (CVL) fluid of women using Eng implants.

- Unlike previous studies, Haddad et al. used lymphocytes recovered from cervicovaginal lavage (CVLs) samples for phenotyping. This is a major limitation because lymphocytes in CVL samples are usually present in low in numbers and have poor viability. In this study, samples with fewer than 100 CD3+ T cells (20%) were excluded from analysis, but actual cell counts were not provided. Flow analysis is not accurate for samples with low cell counts (<20,000 cells). CVL mononuclear cell counts should be provided as the number of HIV target cells is a more meaningful endpoint than % HIV target cells.
- The investigators reported collecting vaginal samples during the luteal and follicular phase of the menstrual cycle which is important because Byrne et al. reported a significant increase in CCR5+ CD4+ cells during the luteal phase. Did the investigators determine whether this was the case in their study before they combined these samples for the analysis?
- Do women using Eng implants have cycling levels of endogenous progesterone and estrogen, and were these included as covariates in the analysis?

Concentrations of cytokines in CVL fluid were also studied, and a modest but statistically significant increase in sCD40L, and decreases in Il-12 and G-CSF were noted. These data were presented as relative percentages (before and after ENG use). It would be helpful to also have the cytokine concentrations to provide a context for the potential physiological significance of these findings.

Since vaginal immune parameters are highly variable and there is no clear pattern of differences in vaginal parameters among women using progestin injections or implants for contraception, the differences in vaginal environment variables described in this report that are attributed to Eng use should be interpreted with caution. Conclusions such as: “Eng implant use led to a moderate increase in the availability of HIV target cells in the genital tract” and “ It is unclear if these implant induced changes would be any less safe than other contraceptives with regard to HIV risk” should be reworded.
